# Assessment of Polymorphism of the VDR Gene and Serum Vitamin D Values in Gestational Diabetes Mellitus 

**DOI:** 10.1055/s-0039-1693678

**Published:** 2019-07

**Authors:** Thais Walverde Siqueira, Edward Araujo Júnior, Rosiane Mattar, Silvia Daher

**Affiliations:** 1 Escola Paulista de Medicina, Universidade Federal de São Paulo, São Paulo, SP, Brazil

**Keywords:** vitamin D, genetic polymorphism, gestational diabetes mellitus, vitamina D, polimorfismo genético, diabetes mellitus gestacional

## Abstract

**Objective** To evaluate the relationship between vitamin D receptor (VDR) gene polymorphism (*FokI* [rs10735810]) and serum vitamin D concentration in gestational diabetes mellitus (GDM).

**Methods** A prospective case-control study that recruited healthy pregnant women (control group) (*n = *78) and women with GDM (GDM group) (*n = *79), with no other comorbidities. Peripheral blood samples were collected in the 3^rd^ trimester of gestation, and all of the pregnant women were followed-up until the end of the pregnancy and the postpartum period. Serum vitamin D concentrations were measured by high-performance liquid chromatography (HPLC). For genomic polymorphism analysis, the genomic DNA was extracted by the dodecyltrimethylammonium bromide/cetyltrimethylammonium bromide (DTAB/CTAB) method, and genotyping was performed by the polymerase chain reaction – restriction fragment length polymorphism (PCR-RFLP) technique, using the restriction enzyme *FokI*. The Student-t, Mann-Whitney, chi-squared, and Fischer exact tests were used for the analysis of the results.

**Results** There was no significant difference between the pregnant women in the control and GDM groups regarding serum vitamin D levels (17.60 ± 8.89 ng/mL versus 23.60 ± 10.68 ng/mL; *p* = 0.1). Also, no significant difference was detected between the *FokI* genotypic frequency when the 2 groups were compared with each other (*p* = 0.41).

**Conclusion** There was no association between the *FokI* polymorphism and the development of GDM, nor was there any change in serum vitamin D levels in patients with GDM.

## Abstract (pt)

**Resumo:**

**Objetivo** Avaliar a relação entre o polimorfismo do gene receptor da vitamina D (VDR) (*FokI* [rs10735810]) e a concentração sérica de vitamina D no diabetes mellitus gestacional (DMG).

**Métodos** Estudo prospectivo tipo caso-controle que recrutou gestantes saudáveis (grupo controle) (*n* = 78) e com DMG (grupo DMG) (*n* = 79), sem outras comorbidades. Foram coletadas amostras de sangue periférico no 3° trimestre da gestação, e todas as gestantes foram acompanhadas até o final da gravidez e no pós-parto. As concentrações séricas de vitamina D foram mensuradas por cromotografia líquida de alta eficiência (CLAE). Para análise do polimorfismo genético, o DNA genômico foi extraído pelo método de brometo de dodeciltrimetilamônio/brometo de cetiltrimetilamônio (DTAB/CTAB), e as genotipagens foram realizadas por técnica de reação de cadeia de polimerase – polimorfismo do comprimento do fragmento de restrição (PCR-RFLP, na sigla em inglês), sendo empregada a enzima de restrição *FokI*. Foram utilizados os testes t-Student, Mann-Whitney, qui-quadrado e exato de Fischer para a análise dos resultados.

**Resultados** Não houve diferença significativa entre as gestantes dos grupos controle e DMG quanto aos níveis séricos de vitamina D (17,60 ± 8,89 ng/mL *versus* 23,60 ± 10,68 ng/mL; *p* = 0,1). Também não foi detectada diferença significativa entre a frequência genotípica de *FokI*, quando comparados os 2 grupos entre si (*p* = 0,41).

**Conclusão** Não foi identificada associação do polimorfismo *FokI* com o desenvolvimento de DMG, bem como não foi observada alteração nos níveis séricos de vitamina D em pacientes com DMG.

## Introduction

Gestational diabetes mellitus (GDM) is the most frequent metabolic disorder of pregnancy. It affects ∼ 3 to 10% of pregnant women, depending on the population studied and on the diagnostic criteria adopted.^1^ ^2^ Gestational diabetes mellitus is diagnosed when glucose intolerance, with consequent hyperglycemia, begins or is only identified during pregnancy,^3^ resulting from the inability of the pancreas to meet the increasing demand for insulin that usually occurs in the 2^nd^ trimester of pregnancy.^3^ ^4^ It is known that GDM is a risk condition both for mother and child, with both immediate and long-term possible complications.^5^

The pathophysiology of GDM is complex and involves the participation of many factors and mechanisms. In addition to insulin resistance, other physiological processes, such as the development of inflammatory response, occur more exacerbatedly in cases of GDM. Thus, while in healthy gestations the inflammatory reaction is mild and transient, in GDM it seems to be more intense and persistent, characterized by hypersecretion of inflammatory cytokines.^6^

In addition to being essential for calcium absorption and bone metabolism, vitamin D is recognized for its anti-inflammatory effect.^7^ It also acts on the mechanism of glucose tolerance and insulin sensitivity. The mechanisms involved in these processes are not yet fully understood, but it is believed that insulin receptor expression increases, thus increasing the insulin response to glucose stimulation. This inverse relationship between vitamin D and the level of insulin resistance has been observed in different clinical conditions, among them diabetes mellitus (DM) II.^8^ ^9^ The role of vitamin D in the pathophysiology of GDM has been investigated in recent years. Although there are controversies, several studies suggest that pregnant women with GDM present reduced levels of this vitamin.^10^ ^11^

Most of the vitamin D receptor (VDR) gene polymorphisms are located in the 3' region of the gene, including the *BsmI*, *ApaI* and *TaqI* variants,^12^ while the *Fokl* polymorphism is located at the 5' end of the gene, near the promoter region.^13^ Some studies suggest the association of these polymorphisms with different clinical conditions, such as in the greater susceptibility to inflammatory^14^ and autoimmune^15^ diseases. To date, only one study has analyzed the relationship between genetic variants of the VDR gene and GDM, showing a positive association between the incidence of the disease and the VDR *FokI* polymorphism.^16^

The participation of vitamin D in the pathophysiology of GDM has not yet been clarified. In addition, little is known about the relationship of polymorphisms related to the genes encoding this vitamin, as well as its receptor and the occurrence of the disease. The joint investigation of these parameters may bring new perspectives that help us to evaluate the risk of a pregnant woman developing GDM.

Therefore, the objective of the present study is to evaluate the relationship between VDR gene polymorphism (*FokI*) and the development of GDM. In addition, we intend to evaluate the correlation between the genotypes and their phenotypic expression, that is, the serum concentration of vitamin D.

## Methods

### Participants

A prospective case-control study was conducted at the Department of Obstetrics of the Universidade Federal de São Paulo (UNIFESP, in the Portuguese acronym), São Paulo, state of São Paulo, Brazil. The present study was approved by the Research Ethics Committee of the UNIFESP, and the women who consented to voluntary participation signed a Term of Consent. The inclusion criteria were: women of reproductive age ≥ 18 years old, singleton gestation with live fetus, gestational age between 28 weeks and 1 day and 33 weeks and 6 days, pregestational body mass index (BMI) ≥ 18.5 kg/m2. The exclusion criteria were: pre-existing DM (I or II); fasting glycemia ≥ 126 mg/dL and/or random glycemia ≥ 200 mg/dL in the current gestation; carriers of pre-existing chronic diseases; carriers of acute infections; transplanted from solid organs; users of steroids, antibiotics, immunosuppressants, antihistamines or anti-inflammatories; and drug users. For the diagnosis of GDM, the recommendation of the International Association of Diabetes and Pregnancy Study Groups (IADPSG) was used, that is, an altered cutoff point in the oral glucose tolerance test (OGTT) of 75 g (fasting > 92–125 mg/dL, 1 hour > 180–199 mg/dL, or 2 hours > 153–199 mg/dL).^17^ All of the participants recruited for the present study were followed-up until the end of the pregnancy. Pregnant women who abandoned prenatal care at the UNIFESP and those who were selected and subsequently presented any obstetric complications were excluded from the analysis. Two groups were set up, matched for race, gestational age and BMI, one with GDM and another as a control group. For the sample size calculation, the power of the test was considered to be 80%, with an alpha error of 5% and a minimum difference of 10% between the groups for a minimum frequency of 11% of the risk allele in the control group. Based on these data, it was estimated that at least 50 participants should be included in each group.

### Blood Collection

A total of 9 ml of blood was collected by venous puncture, 5 mL in a dry tube and 4 mL in an EDTA tube (BD Diagnostics, Franklin Lakes, NJ, USA). The samples were transported in refrigerated thermal bags at a temperature between 2°C and 8°C to the Laboratory of Physiological and Experimental Obstetrics of the Department of Obstetrics of the UNIFESP and were processed up to 4 hours after collection. The DNA extraction sample (collected in the EDTA-containing tube) was centrifuged at 3,000 rpm for 10 minutes at 4°C, and the buffy coat containing the polymorphonuclear cells was collected and stored at - 20°C for further processing. The samples collected in the dry tube were centrifuged after clot retraction at 3,500 rpm for 10 minutes at room temperature. The serum obtained was aliquoted and stored in a sterile and dry microtube at - 80°C for subsequent quantification of Vitamin D, and had a maximum shelf life of 3 months.

### Extraction of DNA

The extraction of genomic DNA was performed by the dodecyltrimethylammonium bromide/ cetyltrimethylammonium bromide (DTAB/CTAB) technique, described by Gustincich et al.^18^ A total of 450 μl of 12% DTAB solution was added to the buffy coat. After incubation at 67°C for 5 minutes, 900 μL of chloroform was added. The sample was centrifuged for 2 minutes at 10,000 rpm, separating the material into 3 phases. The upper phase, with approximately 500 μL, was transferred to a tube with 900 μL of distilled water and 100 μL of 5% CTAB. After being stirred by inversion, the sample formed the precipitate of DNA-CTAB, again being centrifuged at 10,000 rpm for 2 minutes, and the supernatant was discarded. The DNA precipitate was dissolved in 300 μL of 1.2 M NaCl solution and 750 μL of 90% ethanol was added. With stirring by inversion, the DNA was precipitated and centrifuged at 13,000 rpm for 2 minutes, the supernatant was discarded, and the DNA was washed with 70% ethanol, followed by another centrifugation. The supernatant was discarded and the DNA was dissolved in 120 μL of distilled water. The final DNA concentration was adjusted to 100 ng/μL, and was determined by spectrophotometer reading, with absorbance between 260 and 280 nm (UltroSpec III, Pharmacia Biothech, Uppsala, Sweden). The purity of the DNA was confirmed by the ratio A_260nm_/A_280nm_, considering acceptable values between 1.5 and 1.8.

### Polymerase Chain Reaction

Amplifications were performed under standard conditions for polymerase chain reaction (PCR) (Master Mix, Promega Corp., Madison, WI, USA), following the instructions of the manufacturer, using a Peltier Thermal Cycler MJ96G (Biocycle Co. Ltd., Hangzhou, China) with the conditions previously standardized for the primer of interest, according to the specific literature. The PCR-amplified products were analyzed by electrophoresis on 2% agarose gel (Gibco BRL, Paisley, UK) for 45 minutes at ∼ 100 V. Subsequently, they were submitted to the restriction fragment length polymorphism (RFLP) technique, which detects a genetic variation with the use of a restriction enzyme. The products obtained by digestion with restriction enzymes were analyzed by electrophoresis on 3% agarose gel (Gibco BRL, Paisley, UK) for 45 minutes at ∼ 100 V. To analyze the results, we used the E-BOX 300 UV capture system (Vilber Lourmat, Collégien, France).

### Genotyping of Polymorphisms

The selected polymorphism is located in the VDR gene. The amplification of a segment of this region in which the polymorphism is contained was performed with the following cycling: initial denaturation performed with a cycle composed by denaturation at 94°C for 5 minutes, followed by 35 cycles composed by denaturation at 94°C for 30 seconds, annealing at 58°C for 45 seconds, and polymerization at 72°C for 55 seconds. The final extent was at 72°C for 10 minutes. For the analysis of this polymorphism, the sense and antisense primers used were: 5′-AGC TGG CCCTGG CAC TGA CTC TGC TCT-3′ and 5′-ATG GAA ACA CCT TGC TTC TTCTCC CTC-3′, respectively. The FokI restriction enzyme (New England Biolabs Inc., Ipswich, MA, USA) was added to this PCR product.^19^

### Evaluation of Serum Vitamin D Levels

The most indicated technique for assessing vitamin D serum levels is high-performance liquid chromatography (HPLC). 20 The limit of vitamin detection was 1.4 μg/L. The reference values established by the present study were defined as: ideal: between 20.0 and 70.0 ng/mL; insufficient: between 10.0 and 20.0 ng/mL; and deficient: < 10.0 ng/mL.

### Statistical Analysis

The Skewness and Kurtosis, Kolmogorov-Smirnov, and Shapiro-Wilk normality tests were performed to evaluate the distribution of quantitative variables. According to this evaluation, for the comparison of quantitative data, the Mann-Whitney test or the Student t-test for independent samples were applied. The Hardy-Weinberg equilibrium test was applied comparing the expected frequencies of each genotype with the observed values. The chi-squared or the Fisher exact test was used to compare qualitative variables. The significance level was set at 5% (*p* < 0.05).

## Results

First, 174 healthy pregnant women (control group) and 125 pregnant women with GDM (GDM group) were initially recruited. In the GDM group, 46 women were excluded, 1 due to gestational age outside the established interval, 1 due to pregestational BMI ≥ 18.5 kg/m^2^, 3 due to incomplete data, and 41 due to laboratory technical issues. In the control group, 96 women were excluded, 48 due to gestational age outside the established interval, 4 due to pregestational BMI ≥ 18.5 kg/m^2^, 33 due to incomplete data, and 11 due to laboratory technical isssues. Thus, the study included 78 pregnant women and 79 pregnant women with GDM. Table 1 shows the sociodemographic characteristics of the pregnant women evaluated for vitamin D concentration and genotyping of *FokI* polymorphism. We identified a difference in maternal age, which was significantly higher in the group of patients with GDM (33 ± 5.7 versus. 30 ± 6.7 years old; *p* = 0.01).

**Table 1:** Main sociodemographic and clinical characteristics of the analyzed patients

		Control	GDM	*p-value*
Variables		(*n* = 78)	(*n* = 79)	
Age (years old)*	Minimum–maximum	18–43	19–44	0.01**
	Mean	30	33	
	Standard deviation	6.705	5.735	
Gestational age at collection (weeks)*	Minimum–maximum	25 6/7–33 6/7	21 1/7–38 1/7	0.07
	Mean	30 1/7	30 6/7	
	Standard deviation	1.872	2.942	
Pregestational BMI (Kg/m^2^)*	Minimum–maximum	18.50–38.57	18.80–38.20	0.35
	Mean	26.70	25.80	
	Standard deviation	4.448	4.446	
Ethnicity†	White	29 (37.18%)	32 (40.51%)	0.78
	Brown-skinned	47 (60.26%)	46 (58.23%)	
	Black	02 (2.56%)	01 (1.26%)	
Parity†	0	24 (30.77%)	18 (22.78%)	0.50
	1	30 (38.46%)	32 (40.51%)	
	+2	24 (30.77%)	29 (36.71%)	
Smoke¥	Yes	8 (10.26%)	7 (8.86%)	0.79
	No	70 (89.74%)	72 (91.14%)	
Background family (GDM)¥	Yes	14 (17.95%)	21 (26.58%)	0.25
	No	64 (82.05%)	58 (73.42%)	

Abbreviations: BMI, body mass index; GDM, gestational diabetes mellitus.

* Student t-test;

† Chi-squared test;

¥ Fisher exact test

** *p* < 0.05.

Serum vitamin D levels were evaluated in 52 pregnant women in the control group and in 41 patients with GDM. No significant difference was observed between the groups (17.60 ± 8.89 ng/mL versus. 23.60 ± 10.68 ng/mL; *p *= 0.1; for the control and GDM groups, respectively). We did not identify differences in serum vitamin D levels between pregnant controls and GDM patients, regardless of the degree of deficiency presented (Table 2).

**Table 2:** Serum levels of Vitamin D in healthy patients (controls), and in patients with gestational diabetes mellitus

Variables		Control	GDM	*p-value*
Serum levels of vitamin D		(*n* = 52)	(*n* = 41)	
< 10 ng/mL£	***n***	7	3	1.00
	Minimum–maximum	4.400–9.100	6.700–7.900	
	Median	7.50	7.30	
	Standard deviation	1.604	0.6000	
10–20 ng/mL£	***n***	24	14	0.32
	Minimum–maximum	10.40–19.70	10.00–19.20	
	Median	15.70	14.40	
	Standard deviation	2.960	2.614	
20.1–29.9 ng/mL£	***n***	11	12	0.78
	Minimum–maximum	21.10–29.80	20.20–29.60	
	Median	23.50	24.85	
	Standard deviation	3.181	2.833	
≥ 30 ng/mL£	***n***	10	12	0.06
	Minimum–maximum	30.50–36.80	31.40–48.10	
	Median	32.15	35.05	
	Standard deviation	2.343	5.454	

Abbreviation: GDM, gestational diabetes mellitus.

£ Mann-Whitney test.

The evaluation of the *FokI* polymorphism related to the VDR gene was performed in 76 pregnant women and in 72 women with GDM. No significant differences in genotype frequencies were identified separately (*p* = 0.41) or when we grouped to the FF + Ff versus ff (*p* = 0.86), and neither for allele frequencies (*p* = 0.30; odds ratio [OR] = 0.75; confidence interval [CI] = 0.45–1.25) (Table 3).

**Table 3:** Genotypic (FG) and allelic (FA) frequencies of *FokI* polymorphism in control pregnant women with and in women with gestational diabetes mellitus

		Control	GDM	*p-value*
**FG**	FF	37 (48.68%)	39 (54.17%)	0.39†
	Ff	30 (39.47%)	29 (40.28%)	
	Ff	9 (11.85%)	4 (5.55%)	
	FF + Ff	67 (88.16%)	68 (94.45%)	0.25¥
	Total	76	72	
	EWH	*p* = 0.45	*p *= 0.64	
**FA**	F	104	107	0.30¥
	F	48	37	

Abbreviation: GDM, gestational diabetes mellitus.

† Chi-squared test;

¥ Fisher exact test.

As the literature generally defines values < 20 ng/mL as vitamin D deficiency, we have redistributed our patients according to this parameter and reanalyzed the results. Significant differences were not identified for genotypic frequencies analyzed separately (*p* = 0.47), or when we grouped for the FF + Ff standard versus ff (*p* = 0.67), as observed in Table 4.

**Table 4:** Ratio of genotype versus. phenotype (gestational diabetes mellitus + control) according to reference values in the literature

	< 20 ng/mL	≥ 20 ng/mL	*p-value*
**FF**	24 (52.17%)	21 (55.26%)	0.47†
**Ff**	17 (36.96%)	15 (39.47%)	
**Ff**	05 (10.87%)	02 (5.26%)	
**FF** **+** **Ff**	41 (89.13%)	36 (94.73%)	0.67¥
**Total**	46	38	

† Chi-squared test;

¥ Fisher exact test.

## Discussion

Our hypothesis was that pregnant women with GDM had lower levels of vitamin D than controls, and that this imbalance was possibly related to genetic predisposition. However, we did not observe alterations in serum vitamin D levels in patients with GDM when compared with controls. We also did not identify any association between genetic polymorphism of the VDR gene and the occurrence of GDM, nor a correlation between genotype and phenotype.

Recognized for its anti-inflammatory effect and for its role in glucose metabolism, vitamin D has been implicated in the pathophysiology of different clinical conditions, such as obesity and DM II.^20^ ^21^ Vitamin D deficiency appears to have a dose-dependent effect on glucose homeostasis, on insulin secretion, and on insulin resistance. Although controversial, most studies suggest that patients with DM II have deficiency of this vitamin.^22^ ^23^

Regarding gestation, a recent systematic review showed the importance of this vitamin for gestational success, and its deficiency is associated with different obstetric complications.^24^ Reduced levels of vitamin D appear to compromise the physiological adaptation process of the pregnant woman, increasing the degree of inflammation and insulin resistance, preventing the adequate development of the pregnancy. Thus, an association between low vitamin D levels and increased risk of severe preeclampsia and of preterm delivery has been reported.^25^ ^26^ In an obese African-American population, a positive relationship between vitamin D deficiency and GDM was significant only in groups of pregnant smokers.^11^

Due to the limitations of the population attending our service, environmental and ethnic issues were not considered as exclusion or inclusion parameters. Our study group was relatively homogeneous regarding the sociodemographic status of the participants.

The role of vitamin D in the pathophysiology of GDM is controversial.^10^ These controversies are related to several factors, such as the heterogeneity of the studies performed regarding the gestational age at the time of sample collection, the ethnic groups evaluated, differences in the degree of sun exposure (season, geographic region, occupation), among others.^27^ ^28^ ^29^ Age also interferes in the levels of this vitamin due to hormonal changes in women, and because age influences the degree of sun exposure, according to several authors.^30^ ^31^ ^32^

In our study, we have adopted levels of vitamin D ≥ 20 ng/mL as adequate or sufficient, 10 to 20 ng/mL as insufficient, and < 10 ng/mL as deficient. To date, there are no reference values for serum vitamin D throughout gestation. Considering the cutoff points we have set for the present study, about half of the cases included had some degree of vitamin D deficiency, and the controls had lower levels than the GDM group. We believe that limitations in relation to the degree of sun exposure, to the nutritional status or race could have influenced our results.

In the present study, we have evaluated serum samples collected in the 2^nd^ and 3^rd^ trimesters of pregnancy in order to confirm the role of vitamin D in the pathophysiology of GDM. However, most of the studies analyze the predictive value of this dosage for the detection of the patient at risk of GDM.^11^ Therefore, the serum levels in patients < 16 weeks of gestation are analyzed and, later, this data is confronted with the development of the disease. They are different evaluations that can justify the divergences found, even in relation to the prediction.^33^ ^34^ It is also worth noting that, to date, there is no evidence that vitamin D supplementation improves the obstetric prognosis.

Regarding the genetic study, we did not identify an association between the *FokI* VDR polymorphism (rs10735810) and the occurrence of GDM. The only study to date shows a positive association between GDM and the *FokI* polymorphism, but in the Iranian population. In this study, the authors showed that the frequencies of this polymorphism differ between ethnic groups, suggesting that the results obtained in a given population cannot be generalized.^16^ Even the relationship between this polymorphism and DM II is still controversial, probably because of the differences between the sociodemographic, and even the clinical characteristics that exist in the studied populations.

In the present study, no correlation between genotype and phenotype was identified. These relationships are generally observed under restricted technical conditions and in samples with specific characteristics. As limitations, we point out the relatively small number of cases, due to the difficulties faced in selecting participants who fulfilled all the established criteria. As a positive point, it is the first study that evaluated these parameters in a Brazilian pregnant population. The present study included a relatively small number of cases, due to the difficulties faced in selecting participants who met all of the established criteria. The results are preliminary and will need to be validated in future prospective studies with larger samples. Despite these limitations, the present study was a pioneer in the assessment of these parameters in a Brazilian pregnant population.

## Conclusion

In summary, there was no change in serum vitamin D levels in patients with GDM, nor was there an association between the *FokI* polymorphism and the development of GDM. In addition, no relationship was observed between phenotype, serum vitamin D level, and the genotype, genetic variant related to *FokI*.
